# Case Report: Liver and bladder metastasis in a patient with HR-positive, HER2-low breast cancer

**DOI:** 10.3389/fonc.2025.1505354

**Published:** 2025-06-06

**Authors:** Lingting Jiang, Yingkuan Shao, Qiang Chen, Sifeng Tao

**Affiliations:** ^1^ Department of Breast Surgery, Haiyan People's Hospital, Zhejiang, China; ^2^ Key Laboratory of Cancer Prevention and Intervention, Ministry of Education, Department of Breast Surgery and Oncology, Cancer Institute, Second Affiliated Hospital, School of Medicine, Zhejiang University, Zhejiang, Hangzhou, China

**Keywords:** bladder metastasis, HER2 low-expression breast cancer, ds8201a, liver metastasis, bone metastasis

## Abstract

This case report presents a premenopausal patient with high-risk hormone receptor (HR)-positive, HER2-low breast cancer who underwent breast-conserving surgery and axillary lymph node dissection after neoadjuvant chemotherapy. Despite postoperative radiotherapy, the patient did not adhere to endocrine therapy recommendations. Approximately five years later, metastases were identified in the liver, retroperitoneal lymph nodes, bones, and bladder. Notably, bladder metastasis, an exceptionally rare occurrence in breast cancer, was confirmed. Treatment with trastuzumab deruxtecan (T-DXd) demonstrated partial efficacy, though progression-free survival (PFS) was limited to six months. This case underscores the importance of vigilant follow-up and consideration of rare metastatic sites as breast cancer treatments evolve and improve. Bone-targeted therapies played a critical role, but the patient’s short PFS suggests significant tumor heterogeneity. This report discusses a rare case of bladder metastasis originating from HR-positive, HER2-low breast cancer, emphasizing innovative treatments such as T-DXd.

## Introduction

Breast cancer is the most common malignancy and the leading cause of cancer-related deaths among women worldwide (1). The most common sites of metastasis for breast cancer typically include the bones, liver, lungs, and brain (2), reports of bladder metastasis are limited. In contrast to these more commonly affected areas, the urinary bladder emerges as an unusual site for the development of distant metastasis. In some cases, bladder metastasis originating from breast cancer accounted for only 2.5% of secondary bladder tumors (3). With the introduction of various innovative therapies, patients with advanced breast cancer now have more treatment options. This report describes a case of bladder metastasis originating from breast cancer, where multiple innovative drugs, such as T-Dxd (4), were employed, leading to favorable outcomes.

## Case description

**Figure 1:**
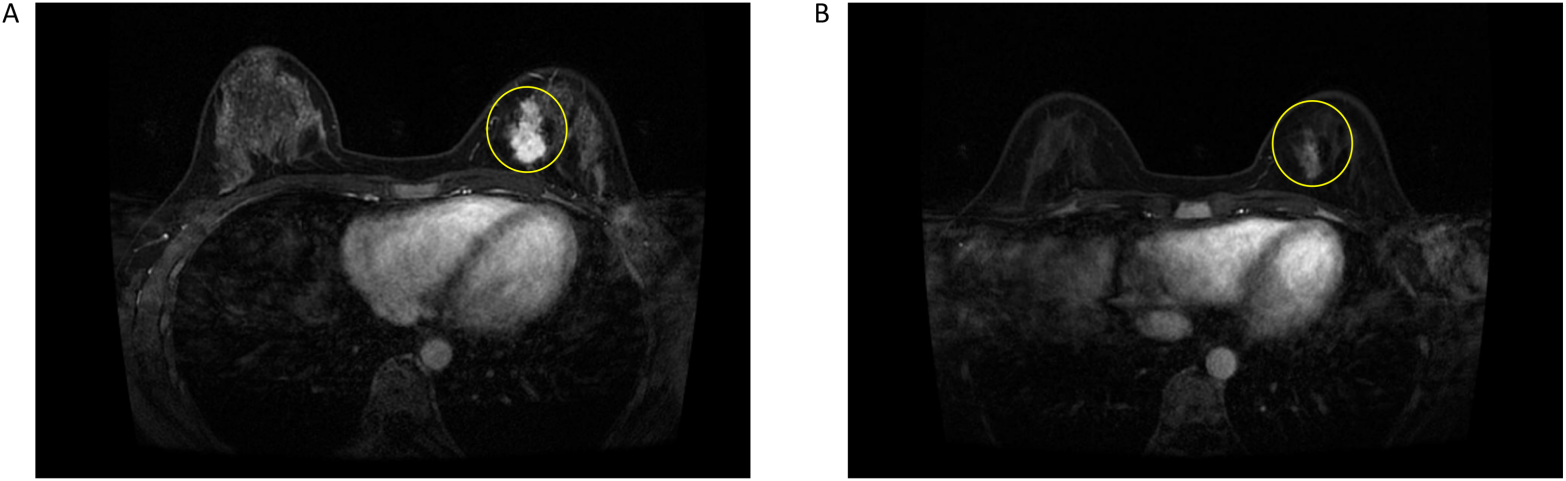
**(A)** Breast enhanced MRI before neoadjuvant chemotherapy. **(B)** Breast enhanced MRI after neoadjuvant chemotherapy.

**Figure 2:**
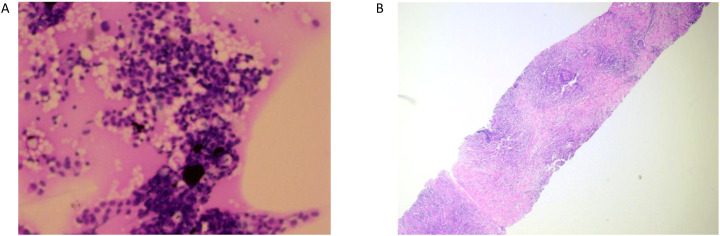
**(A)** Left axillary lymph node puncture pathology picture. **(B)** Left breast mass puncture pathology picture.

**Figure 3:**
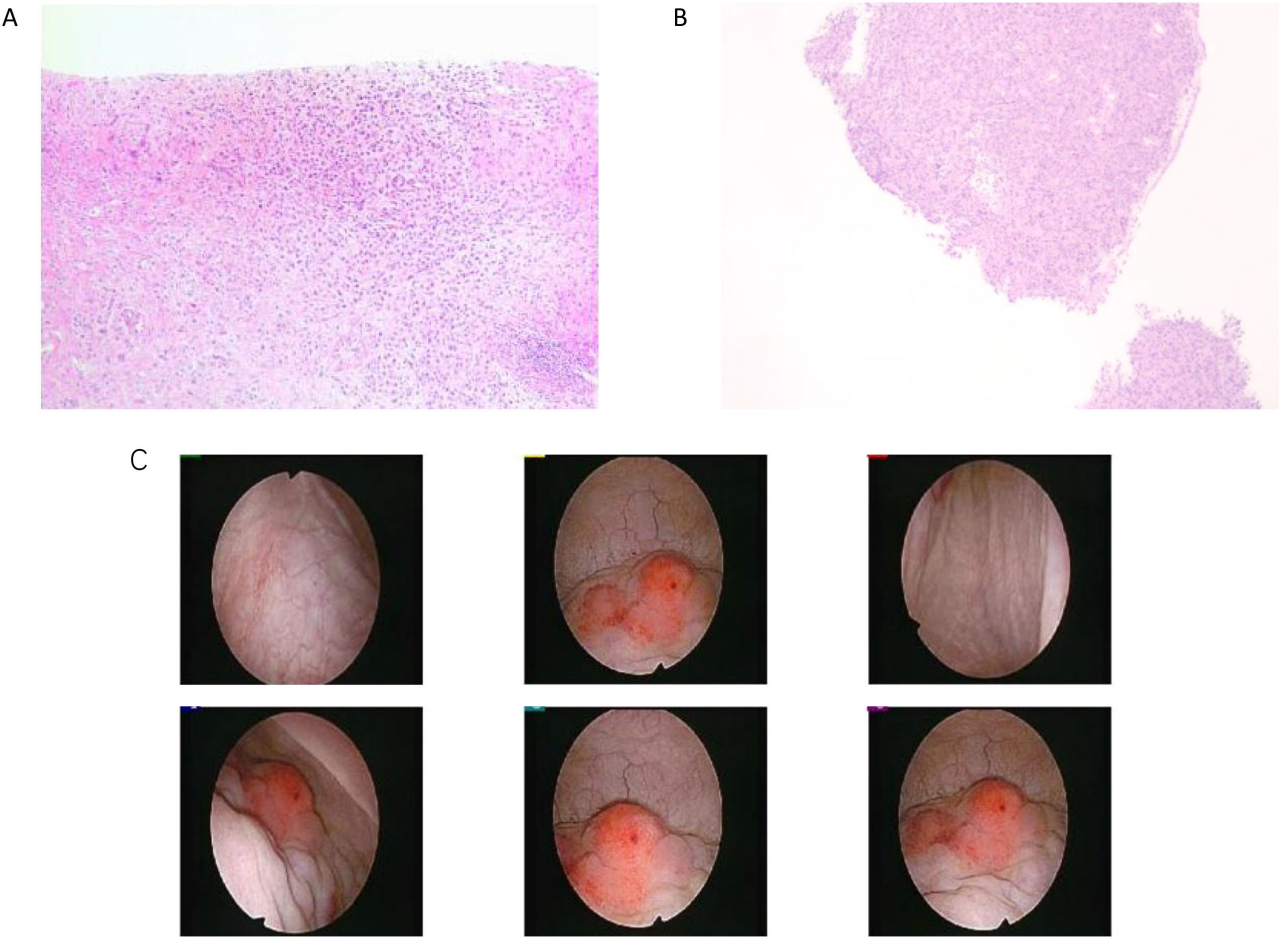
**(A)** Pathological image of liver puncture. **(B)** Pathological biopsy image of cystoscopy. **(C)** Normal Bladder mucosa and vascular texture: the posterior wall of the bladder was thickened and thickened near the top mucosa, the texture was about 3*4cm, and biopsy was performed. Muscle trabeculae: not seen. Ureteral orifice spurting and urine color: visible on both sides, urine sprayed clear.

**Figure 4:**
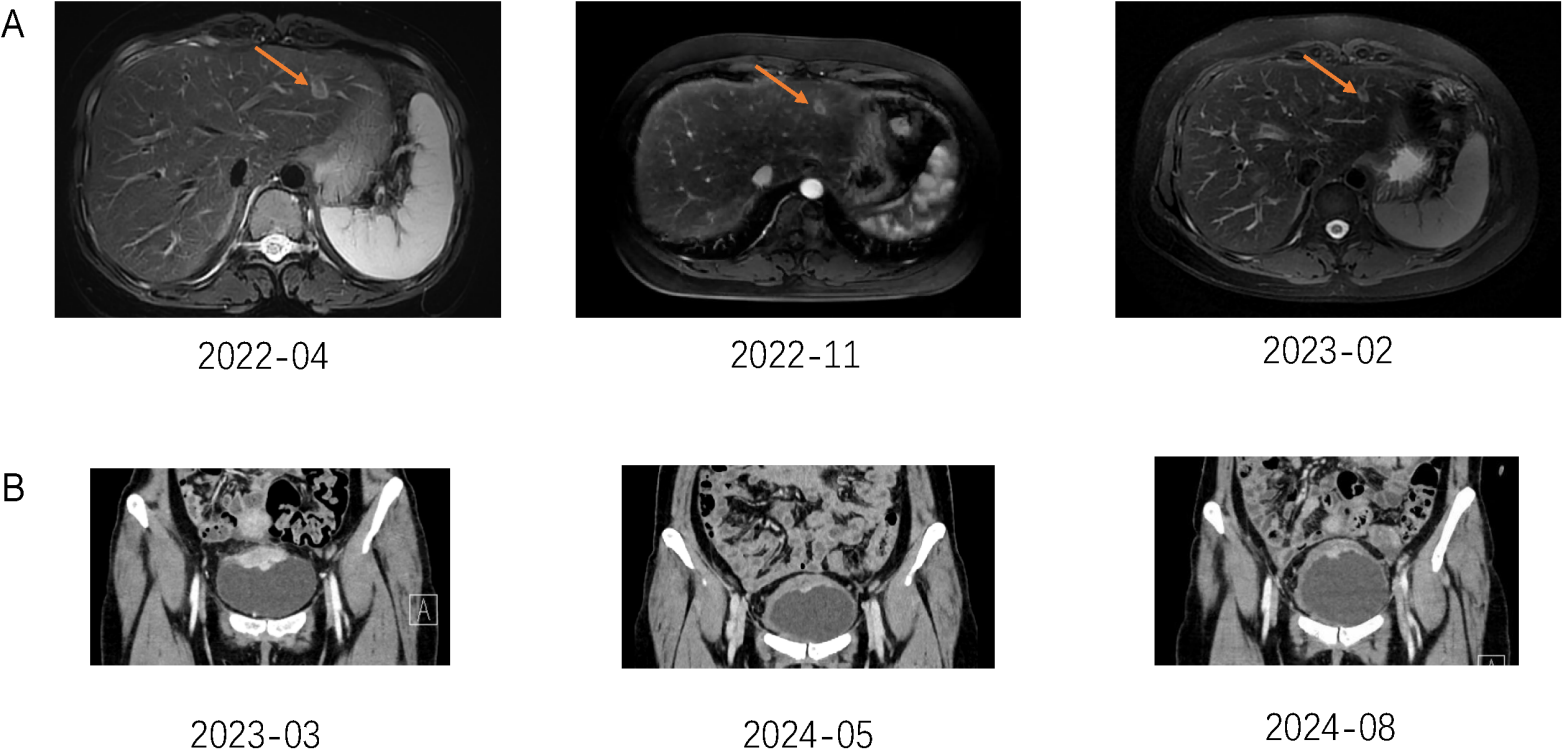
**(A)** The Orogen arrow in the figure shows liver metastases. **(B)** Pelvic MRI examination results suggest that bladder metastases have shrunk.

A 31-year-old Asian woman was diagnosed with breast cancer with axillary lymph node metastasis based on core needle biopsy. She had no family history of cancer and was otherwise healthy. Physical examination revealed a 3x2 cm palpable, hard, unmovable, and non-tender mass in the outer lower quadrant of the left breast, along with enlarged, firm axillary lymph nodes. Ultrasound examination showed a hypoechoic mass (~3.2×1.5 cm) in the lower outer quadrant of the left breast (near the border of the inner and outer quadrants), while MRI identified a lobulated mass in the lower inner quadrant, classified as BI-RADS 4C (**Figure 1A**). Core needle biopsy of the left breast mass confirmed invasive carcinoma (**Figure 2A**). Immunohistochemistry (IHC) revealed: ER 70% (2-3+), PR 30% (2-3+), HER2 (c-erbB-2) 0, Ki-67 10%, CK5/6-, E-Cadherin+, P120 membrane+, and no evidence of vascular invasion. Axillary lymph node biopsy showed metastatic carcinoma (**Figure 2B**) with IHC: ER 90% (3+), PR 90% (3+), HER2 (c-erbB-2) 1+, Ki-67 20%, and AR 90% (3+). Further staging workup, including chest CT, abdominal CT with contrast, brain MRI, abdominal ultrasound, and bone scan, showed no evidence of distant metastasis. The patient underwent neoadjuvant chemotherapy from December 2016 to May 2017, consisting of eight cycles of EC (epirubicin + cyclophosphamide) followed by T (docetaxel) chemotherapy, with concurrent ovarian function suppression (OFS) for ovarian protection. After neoadjuvant treatment, the breast tumor’s largest diameter decreased from approximately 3 cm to 2 cm, consistent with a partial response (**Figure 1B**). In June 2017, the patient underwent breast-conserving surgery with axillary lymph node dissection. The surgical specimen revealed residual invasive carcinoma (2 x 1 cm, WHO grade II) and ductal carcinoma *in situ* (DCIS) involving 30% of the tumor area. Lymphovascular invasion was not detected. The surgical margins were negative, and 2 out of 14 axillary lymph nodes were positive for metastatic carcinoma. Postoperative pathological evaluation showed that the efficacy of neoadjuvant therapy was Miller & Payne (MP) grades 3. Post-surgical IHC of the residual tumor showed: ER 90% (3+), PR 70% (2-3+), HER2 (c-erbB-2) 1+, Ki-67 5%, AR 90% (3+), GATA-3 +, E-Cadherin +, P120 membrane +, and no evidence of vascular invasion. The lymph node metastasis was ER 90% (3+), PR-, HER2 (c-erbB-2) 2+, Ki-67 10%, and AR 90% (3+). FISH testing for HER2 was negative. Based on these findings, the post-neoadjuvant pathological stage was ypT1N1M0. The patient received adjuvant endocrine therapy with OFS (GnRH agonist) plus tamoxifen; however, she elected to discontinue OFS in December 2018 (after ~1.5 years) and continued on tamoxifen alone. In March 2021, upon resumption of menses, OFS was briefly restarted, but the patient ultimately stopped OFS permanently later that year by her own decision. In February 2022, the patient presented with irregular vaginal bleeding and was diagnosed with endometrial polyps after hysteroscopic resection. In April 2022, MRI of the liver revealed multiple hypervascular nodules, with the largest measuring 14x10 mm in segment II, suggestive of metastatic disease. A PET/CT scan showed hypermetabolic retroperitoneal lymph nodes and multiple bone metastases. Liver biopsy confirmed metastatic carcinoma consistent with the breast cancer origin based on IHC: CK (AE1/AE3)+, ER 95% (2-3+), PR 95% (2-3+), AR 95% (3+), HER2 (c-erbB-2) 0, Ki-67 10%+, GATA-3+, and SOX10- (**Figure 3A**).The patient was started on OFS (GnRH agonist) + AI (exemestane) + CDK4/6 inhibitor (abemaciclib) + bone-targeting agents (zoledronic acid) as first-line treatment for HR+ HER2- metastatic breast cancer. Follow-up imaging review in August 2022, November 2022, and February 2023 showed stable disease (**Figure 4A**).

In March 2023, the patient developed hematuria and was diagnosed with bladder metastasis based on cystoscopy (**Figure 3B**) and biopsy, with IHC findings indicating breast cancer origin (**Figure 3C**): ER 90% (1-2+), PR <1%, HER2 (c-erbB-2) 2+, Ki-67 35%, AR 95% (3+), GATA-3+, CK (AE1/AE3)+, and P63-. FISH testing for HER2 was negative. PET/CT in April 2023 showed disease progression, including new bladder lesions and increased metabolic activity in retroperitoneal lymph nodes and bones. With the newly diagnosed bladder metastasis and the overall progression of the disease, the patient was advised to consider chemotherapy, as there was no BRCA mutation. However, the patient refused chemotherapy, leading us to switch from AI to Fulvestrant and change the CDK4/6 inhibitor (Ribociclib) according to the post-MONARCH study, though with limited benefit (5). In June 2023, the patient experienced increased pain and bloating in the lower abdomen. An abdominal ultrasound revealed hydronephrosis of the upper left ureter and hydronephrosis of the left renal pelvis. After visiting the urology department, a “bilateral ureteral stent placement through the urethra” was performed. Given the disease progression and HER2 2+ status of the bladder metastasis (**Table 1**), the patient was offered T-DXd based on the DB-04 trial results (6). HER2 expression of this patient was heterogeneous (ranging from IHC 0 in the initial biopsy to IHC 1+ in the residual tumor and 2+ in a lymph node and bladder lesion, all with negative FISH), consistent with an overall HER2-low phenotype. Review evaluation PR in 2023–10 during treatment period, however, the review in 2024–02 showed progressive disease (PD) of pelvic area and PR of liver. MRI of pelvic showed the inner wall of the left triangular area, and the middle and upper rectum have become larger than before. The appendages on both sides are full and progressive, and metastasis is possible. No obvious liver metastases were found. The patient had not undergone gene sequencing of bladder metastases, and had progressed in endocrine and ADC drug treatment. Given the patient’s rapid disease progression and severe symptoms, which made tolerance difficult, and the failure of multiple lines of therapy, nab-paclitaxel was selected as the patient had not received chemotherapy post-surgery. The regimen, implemented in January 2024, was 400 mg of nab-paclitaxel administered once every three weeks.

**Table 1:** Summary of HER2 expression in different metastatic sites.

Location	HER2 expression (IHC)	HER2 amplification status (FISH)
Breast	0	NA
Axillary lymph nodes	1+	NA
Liver metastasis	0	NA
Bladder metastasis	2+	Negative

During the chemotherapy period, routine assessment checks were performed. In May 2024, an abdominal assessment revealed moderate hydronephrosis in the left kidney. After an ultrasound-guided evaluation, a nephrostomy was deemed feasible and was performed on June 17, 2024, under ultrasound guidance. Since the initiation of nab-paclitaxel chemotherapy, the patient has experienced significant improvements in symptoms of fatigue, loss of appetite, and lower abdominal bloating. A pelvic MRI assessment showed that the metastatic lesions in the anterior superior wall of the bladder, the inner wall of the left trigone area, and the middle to upper segment of the rectum had slightly reduced in size. The monotherapy with nab-paclitaxel has been notably effective, achieving PR (**Figure 4B**).

## Discussion

Bladder metastasis from breast cancer is extremely rare, with approximately 39% of such cases initially presenting with hematuria as the main symptom (7, 8).This case highlights the rarity of bladder metastasis in breast cancer patients and the importance of considering unusual metastatic sites in patients with metastatic breast cancer. In this case, bilateral ureteral stents were placed to relieve obstruction caused by the bladder metastasis, exemplifying the use of local therapy for symptom palliation in metastatic sites, aside from such palliative measures, no surgical resection of the bladder tumor was performed due to the presence of widespread disease, and management followed the principles of systemic therapy. To date, there have been no reports on the use of T-DXd for bladder metastasis in HER2 2+ breast cancer. Based on the results from DB04, we treated the patient and achieved a progression-free survival (PFS) of six months. Mosele et al. in the DASISY Phase 2 study found that the anticancer efficacy of T-DXd significantly increases with higher levels of HER2 expression (9). They also observed that T-DXd can be absorbed and utilized by tumor cells even with minimal HER2 expression, potentially broadening the population that benefits from T-DXd. In the challenging and complex field of HR-positive, low HER2-expressing breast cancer, T-DXd is emerging as a novel therapeutic option. HER2 expression in HR-positive, HER2-low breast cancer is often heterogeneous, manifesting both spatial diversity (coexisting subclones or lesions with different HER2 levels) and temporal variability (10). Mechanistically, this heterogeneity may arise from clonal evolution and selective pressure from therapies (e.g. prolonged endocrine treatment), leading to divergent HER2-expressing populations within the same tumor. Such intra-tumoral and inter-lesional heterogeneity can compromise the uniform efficacy of HER2-targeted therapies, as discordant HER2 expression has been linked to treatment resistance or failure (11). Notably, novel antibody–drug conjugates like trastuzumab deruxtecan (T-DXd) have demonstrated substantial efficacy in HER2-low tumors (12), but their benefit could be limited if significant tumor fractions lack HER2 expression. Clinically, heterogeneity in HER2-low disease underscores the importance of re-evaluating HER2 status at recurrence or progression. Approximately 30–40% of patients exhibit a switch between HER2-low and HER2–0 from primary tumor to relapse, and those acquiring HER2-low expression upon relapse may gain sensitivity to HER2-targeted therapies. Miglietta et al. analyzed 547 matched primary and relapsed breast cancers and found an overall HER2 status discordance of 38%. This was mainly due to tumors switching from HER2–0 to HER2-low (15% of cases) and from HER2-low to HER2-0 (14%) (13). Lin et al. reported on a large cohort of 1,299 breast cancer patients from Fudan University (China) with HER2 status documented in both primary and recurrent/metastatic samples. They observed HER2 status conversion in 28.5% of patients. Specifically, among primary HER2–0 tumors, about one-third (31.7%) converted to HER2-low at recurrence (and a small subset ~3% converted to HER2-positive) (10). Anderson et al. examined 171 paired primary and metastatic tumors and similarly found that roughly one-third (31.7%) of patients showed conversion between HER2-low and HER2–0 status. In this U.S. cohort, loss of HER2-low expression was more common than gain: conversion from HER2-low to HER2–0 occurred in 43% of HER2-low primaries, whereas conversion from HER2–0 to HER2-low occurred in ~23% of HER2–0 primaries (14). For patients progressing on CDK4/6 inhibitors, targeted therapies or alternative endocrine therapies may offer limited benefits. Post-MONARCH study findings suggest a potential role for abemaciclib combined with fulvestrant as second-line therapy, though the absolute benefit remains limited (15, 16). Metastasis to the liver and bladder is an uncommon occurrence in HR-positive, HER2-low breast cancer, underscoring the complexity of metastatic breast cancer (MBC). The second-line treatment options for advanced HR-positive, HER2-negative breast cancer are critical in managing patients who progress after first-line therapies. Common therapeutic strategies include the use of CDK4/6 inhibitors in combination with endocrine therapy. Evidence suggests that this approach significantly improves progression-free survival (PFS) and overall survival (OS) in this patient population (17, 18). In the context of liver metastasis, hepatic resection may be considered in select cases, particularly when systemic disease is well-controlled (19). The role of locoregional treatments, such as radiofrequency ablation or transarterial chemoembolization (TACE), is evolving, offering potential benefits in controlling liver-dominant disease (20–22). Bladder metastasis from breast cancer is an exceptionally rare event, with few cases documented in the literature. The clinical presentation can mimic primary bladder cancer, and diagnosis is often established through histopathological evaluation (23). No additional next-generation sequencing or broad genomic profiling was conducted on the tumor; this is a limitation of our report, as such analysis might have identified actionable mutations (for example, PIK3CA or ESR1 mutations) to further guide therapy. Management typically follows the principles of systemic treatment for metastatic breast cancer, though local treatment modalities may be required to alleviate symptoms (24).This case emphasizes the need for a multidisciplinary approach in managing complex metastatic patterns in breast cancer. Personalized treatment plans that incorporate systemic therapy, surgical intervention, and local treatments are essential in optimizing outcomes for patients with advanced HR-positive, HER2-low breast cancer.

## Conclusion

Despite aggressive treatment, the patient exhibited progression in the bladder metastasis while achieving partial response in liver metastasis. Bladder metastasis from breast cancer is rare but requires close monitoring and may benefit from targeted therapies based on molecular profiling. The presented case highlights the rare occurrence of liver and bladder metastasis in HR-positive, HER2-low breast cancer, contributing valuable insights into the metastatic behavior and therapeutic strategies for this breast cancer subtype. Further research is warranted to better understand the mechanisms driving unusual metastatic patterns and to optimize treatment protocols for improved patient outcomes.

## Data Availability

The original contributions presented in the study are included in the article/supplementary material. Further inquiries can be directed to the corresponding author.
